# *Sphagnum* Mosses - Masters of Efficient N-Uptake while Avoiding Intoxication

**DOI:** 10.1371/journal.pone.0079991

**Published:** 2014-01-09

**Authors:** Christian Fritz, Leon P. M. Lamers, Muhammad Riaz, Leon J. L. van den Berg, Theo J. T. M. Elzenga

**Affiliations:** 1 Department of Aquatic Ecology and Environmental Biology, Radboud University Nijmegen, Nijmegen, The Netherlands; 2 Department of Environmental Science, GC University, Faisalabad, Pakistan; 3 Centre for Energy and Environmental Studies, University of Groningen, Groningen, The Netherlands; 4 Environment Department, University of York, York, United Kingdom; 5 Laboratory of Plant Physiology, University of Groningen, Groningen, The Netherlands; WSL Institute for Snow and Avalanche Research SLF, Switzerland

## Abstract

Peat forming *Sphagnum* mosses are able to prevent the dominance of vascular plants under ombrotrophic conditions by efficiently scavenging atmospherically deposited nitrogen (N). N-uptake kinetics of these mosses are therefore expected to play a key role in differential N availability, plant competition, and carbon sequestration in *Sphagnum* peatlands. The interacting effects of rain N concentration and exposure time on moss N-uptake rates are, however, poorly understood.

We investigated the effects of N-concentration (1, 5, 10, 50, 100, 500 µM), N-form (^15^N - ammonium or nitrate) and exposure time (0.5, 2, 72 h) on uptake kinetics for *Sphagnum magellanicum* from a pristine bog in Patagonia (Argentina) and from a Dutch bog exposed to decades of N-pollution.

Uptake rates for ammonium were higher than for nitrate, and N-binding at adsorption sites was negligible. During the first 0.5 h, N-uptake followed saturation kinetics revealing a high affinity (K_m_ 3.5–6.5 µM). Ammonium was taken up 8 times faster than nitrate, whereas over 72 hours this was only 2 times. Uptake rates decreased drastically with increasing exposure times, which implies that many short-term N-uptake experiments in literature may well have overestimated long-term uptake rates and ecosystem retention. *Sphagnum* from the polluted site (i.e. long-term N exposure) showed lower uptake rates than mosses from the pristine site, indicating an adaptive response.

*Sphagnum* therefore appears to be highly efficient in using short N pulses (e.g. rainfall in pristine areas). This strategy has important ecological and evolutionary implications: at high N input rates, the risk of N-toxicity seems to be reduced by lower uptake rates of *Sphagnum*, at the expense of its long-term filter capacity and related competitive advantage over vascular plants. As shown by our conceptual model, interacting effects of N-deposition and climate change (changes in rainfall) will seriously alter the functioning of *Sphagnum* peatlands.

## Introduction

Bogs (ombrotrophic peatlands, i.e. predominantly fed by rainwater, from Greek *ombros*, rain, and *trephein*, to feed) are exceptional ecosystems that may show high storage rates for nutrients and carbon, while nutrient availability is very low due to ombrotrophic conditions (water and nutrient input solely by rain) limiting the growth of vascular plants. A set of traits, unique to *Sphagnum* mosses, enable their dominance in bog ecosystems. *Sphagnum* has evolved a high nutrient use efficiency to cope with low input rates of nutrients [1], [2]. The atmospheric input of nutrients is efficiently retained in moss peat and decomposition rates are low, due to the high retention of rainwater, acidic conditions and poorly degradable organic matter [3]. In addition, a substantial part of the C losses (including CH_4_) are refixed by *Sphagnum* as growth and photosynthesis have been shown to increase upon elevated CO_2_ in porewater [4]–[6]. This combination of traits enables *Sphagnum* to avoid being outcompeted by vascular plants. However, increased availability of nitrogen, e.g. by high airborne inputs, favours vascular plants at the expense of *Sphagnum* mosses. Displacement of *Sphagnum* by vascular bog plants often leads to reduced storage of nutrients, carbon (peat) and water [7]–[9]. In comparison to vascular plants, *Sphagnum* spp. show low decomposition rates due their chemical composition [2], [3], in addition to anoxic and acidic conditions, and may still have similar primary production rates [10], [11]. *Sphagnum* peat may therefore accumulate substantial amounts of nutrients, if considered m^−2^ y^−1^, even though nutrient concentrations in *Sphagnum* are lower.

Bog vegetation hardly responds to small increments of nitrogen availability as the living *Sphagnum* layer filters nitrogen by retaining and storing substantial amounts of nitrogen in biomass and peat [12], [13]. This nitrogen filter prevents the build-up of airborne nitrogen and increased N availability in soil layers below the moss carpet. Cryptogams in general appear to be more efficient in retaining atmospheric nitrogen than other plant groups [14], [15]. Moss biomass has even been found to be the major determinant of N retention capacity of ecosystems [16], [17]. Therefore, mosses are thought to dampen effects of increased anthropogenic inputs of nitrogen [18], [19].

High nitrogen deposition rates were, however, found to have detrimental effects on biomass production of mosses [20], [21]. Negative effects of nitrogen can be direct (e.g. lower photosynthesis; increased metabolic costs) and indirect (e.g. shading and lower water availability due to increased cover of vascular plants). Direct physiological changes in mosses including *Sphagnum* have also been found upon increased availability of nitrogen [1], [22], [23]. High uptake rates can cause a saturation of nitrogen in *Sphagnum* mosses [1], [12], which is often followed by growth reduction [24], [25]. A meta-analysis suggested a general decline in biomass production of *Sphagnum* mosses with increasing nitrogen tissue content [21]. At present, however, little is known about mechanism causing negative effects of nitrogen (saturation) at the cellular level.

Indirect effects of nitrogen are mostly related to an increased cover of vascular plants and concomitant shading of the *Sphagnum* layer [26], [27]. Vascular plants can also lower water availability to *Sphagnum* mosses by increased ecosystem evaporation resulting in lower water levels. Leaching of nitrogen through the moss layer plays a major role in the vegetation change by increasing the availability of N in the rhizosphere to vascular plants [26], [28]. As a dense cover of vascular plants has the potential to decrease moss biomass, vascular plants may also indirectly increase nitrogen leaching and availability in the root zone, as has been suggested by field studies [17], [29]. Leaching of nitrogen in bogs is also facilitated by the high porosity of the living *Sphagnum* layer and underlying layers of litter and recently formed peat.

Water from low-intensity rainfall may have an average residence time of 10–30 min in the *Sphagnum* layer [30]–[32]. Excessive rainfall >5 mm may remain for only 0.5–5 min in the uppermost *Sphagnum* layer [32] (Fritz pers. observation). Future scenarios for rainfall predict increases in excessive rain events in regions with a substantial cover *Sphagnum* peatlands [33], possibly leading to higher leaching of N. Leaching of nitrogen may also occur when nitrogen uptake efficiency of mosses is decreased. Earlier studies found that nitrogen uptake can be induced by high pulses of nitrogen and seems to be variable in time [1], [34]. A slowing down of nitrogen uptake may result from nitrogen saturation at the cellular level [12], [35]. Alternatively, metabolic changes (e.g. amino acid formation, accumulation of free ammonium) may slow down nitrogen uptake.

Several studies have shown that prolonged exposure to elevated nitrogen deposition can result in a decreased uptake efficiency and nitrogen use efficiency for mosses [36], [37] and vascular plants [38]. In contrast, mosses from sites in Sweden with elevated nitrogen deposition showed higher uptake rates of inorganic nitrogen than mosses from a low deposition site [39]. Considering these contradictory results, a better understanding of the nitrogen uptake kinetics of *Sphagnum* mosses seems to be necessary to predict nitrogen retention time and leaching potential at elevated atmospheric nitrogen deposition and its interaction with hydrology (e.g. residence time of rain).

In this study we address the question whether increased exposure time to elevated nitrogen inputs (ammonium or nitrate) results in a reduction of nitrogen uptake efficiency and whether this can be related to nitrogen saturation. We therefore conducted a series of uptake experiments with the peat-forming species *Sphagnum magellanicum* using ^15^N-ammonium and ^15^N-nitrate, which mark the upper and lower ranges of N-concentrations for inorganic and organic nitrogen forms in rain, respectively. Long-term effects of nitrogen exposure were assessed by comparing uptake rates of *Sphagnum* mosses collected from a pristine site in Patagonia (low N deposition 1–2 kg N ha^−1^ y^−1^) and N-polluted site in the Netherlands (N deposition 20 to 40 kg N ha^−1^ y^−1^ over the period 1980–2010). It was hypothesised that historic exposure time, as influenced by weather conditions and climate, interact with concentration effects, and that acclimatization to high N affects N-uptake kinetics. The kinetic parameters for nitrogen uptake by *Sphagnum* plants from different sites were used to model the potential leaching of nitrogen to deeper soil layers, thereby becoming available to roots of vascular plants and increasing their competitive strength.

## Materials and Methods

### Ethical statement

For the Dutch site/N-polluted site: TJTM Elzenga received the sampling permit and samples from Mr. Albert Henckel of the Dutch National Forest Service that manages the National Park Dwingelderveld. In that case permits are usually not issued in a written form in the first place.

For the Argentinean site/pristine site: No specific permits were required for the described field study. The site was not privately-owned land or was protected at the moment of sampling. *Sphagnum magellanicum* is not endangered or protect species in Argentina. The sampling site is under supervision of the provincial government. Transport permission of the samples was 091429.

To the author's knowledge an ethical statement was not needed for the mosses that were inlcuded in the present study.

### Study site, climate and N-deposition

**Figure 1:**
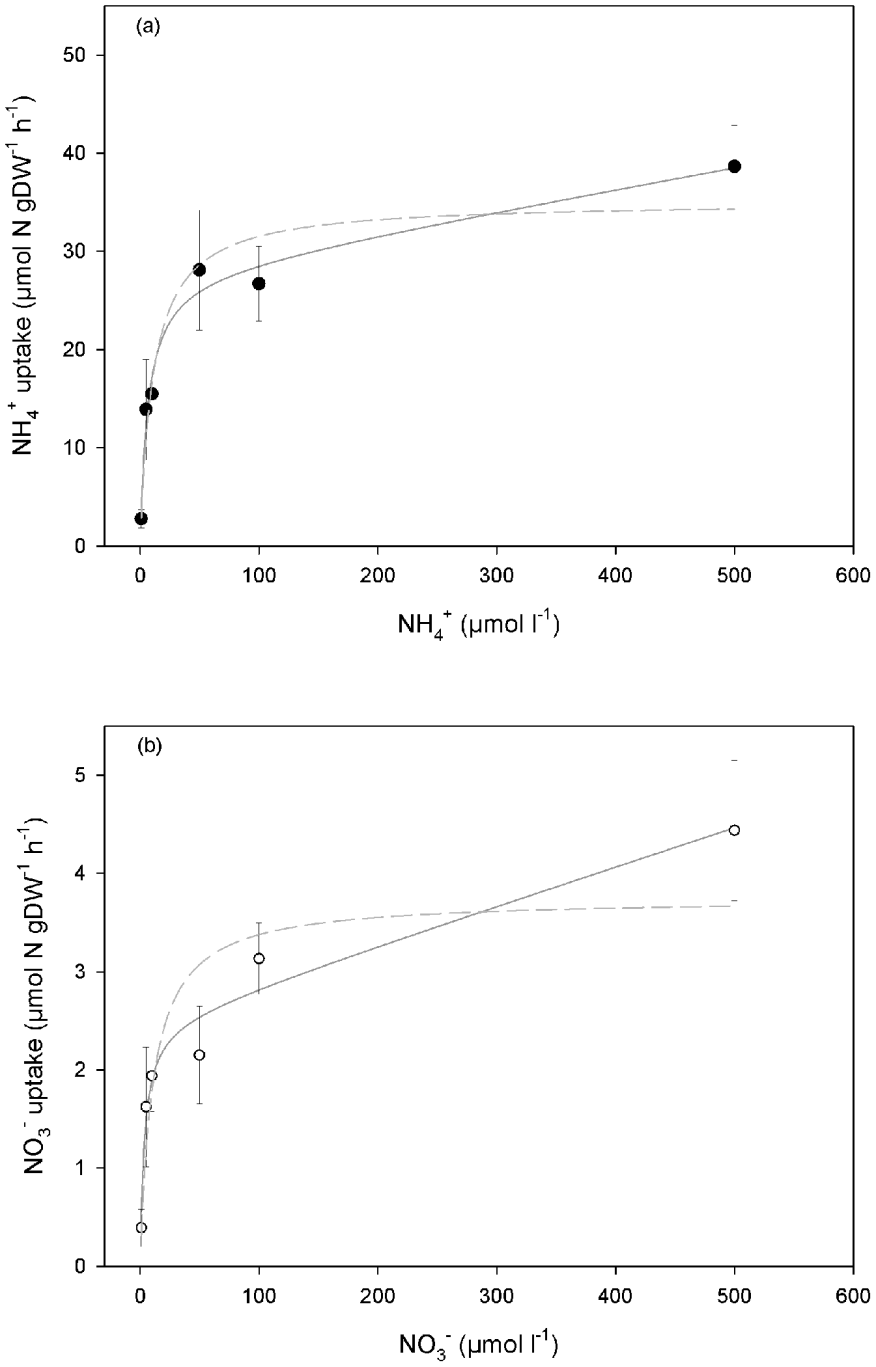
Dose response curves of (a) ammonium uptake rates (filled circles; µmol NH_4_^+^ gDW^−1^ h^−1^) and (b) nitrate uptake rates (open circles; µmol NO_3_^−^ gDW^−1^ h^−1^) of *Sphagnum magellanicum* (*n* = 25) from the pristine site. The rates represent the average uptake rates after 0.5(*n* = 5). Note that y-axes differ by one order of magnitude. Saturation curves (Eq. 1) were fitted to rates: V_max_ of ammonium (28 µmol NH_4_^+^ gDW^−1^ h^−1^) was higher than V_max_ of nitrate 2.5 µmol NO_3_^−^ gDW^−1^ h^−1^), whereas K_m_-values were similar (6.5 µM for ammonium and 3.5 µM for nitrate, respectively). The linear component k was higher for ammonium (0.022 l g^−1^DW h^−1^) than for nitrate (0.004 l g^−1^DW h^−1^). Dashed lines show the fit to uptake measurements using only two parameters (V_max_ and K_m_).

**Figure 2:**
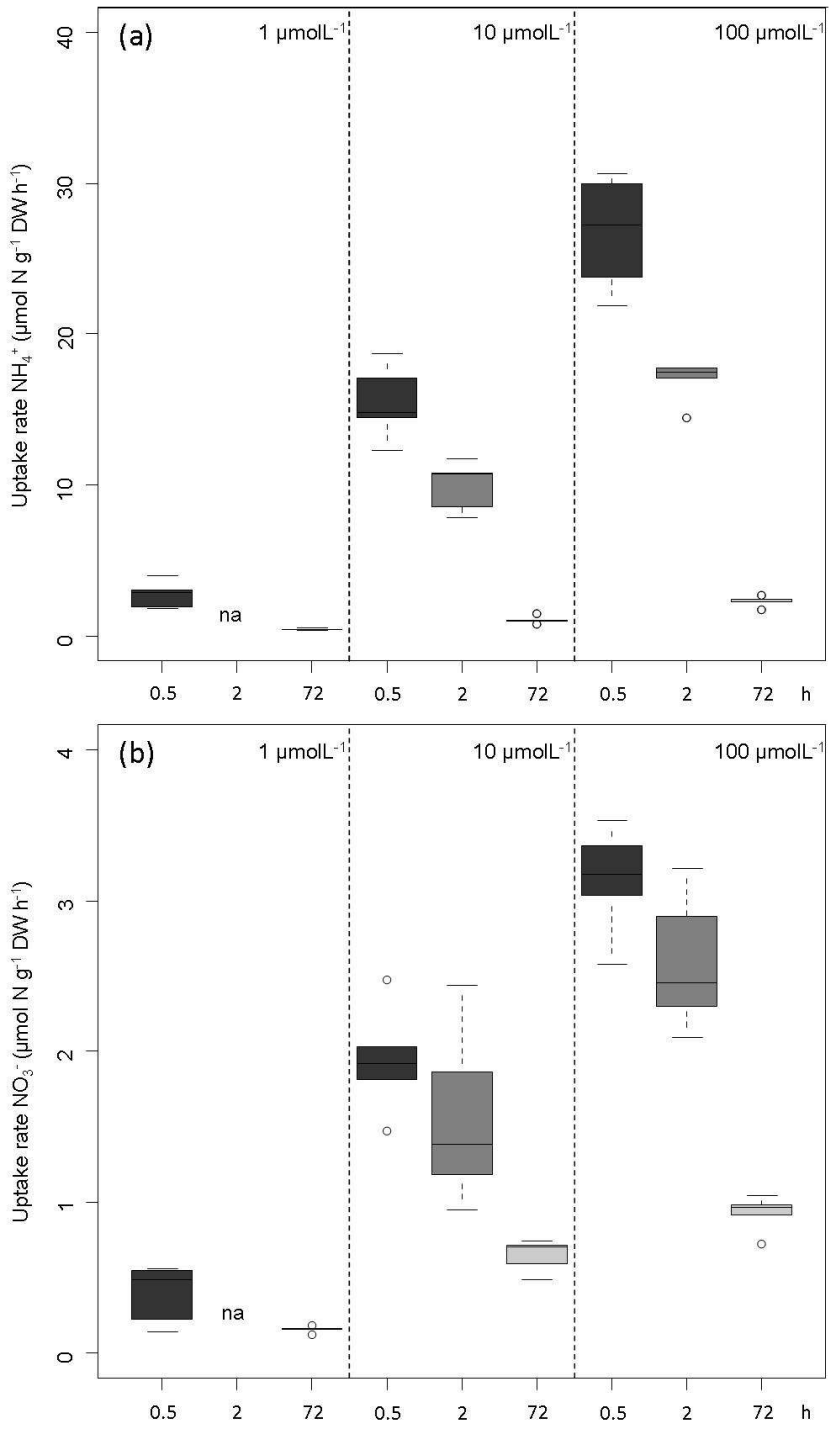
Average uptake rates (µmol N gDW^−1^ h^−1^) after 0.5 (dark grey), 2 (grey) and 72 (light grey) hours, respectively in *Sphagnum magellanicum* (*n* = 5) from the pristine site (Argentina). The upper panel (a) shows ammonium (NH_4_^+^) uptake, the lower panel (b) nitrate (NO_3_^−^) uptake.

**Figure 3:**
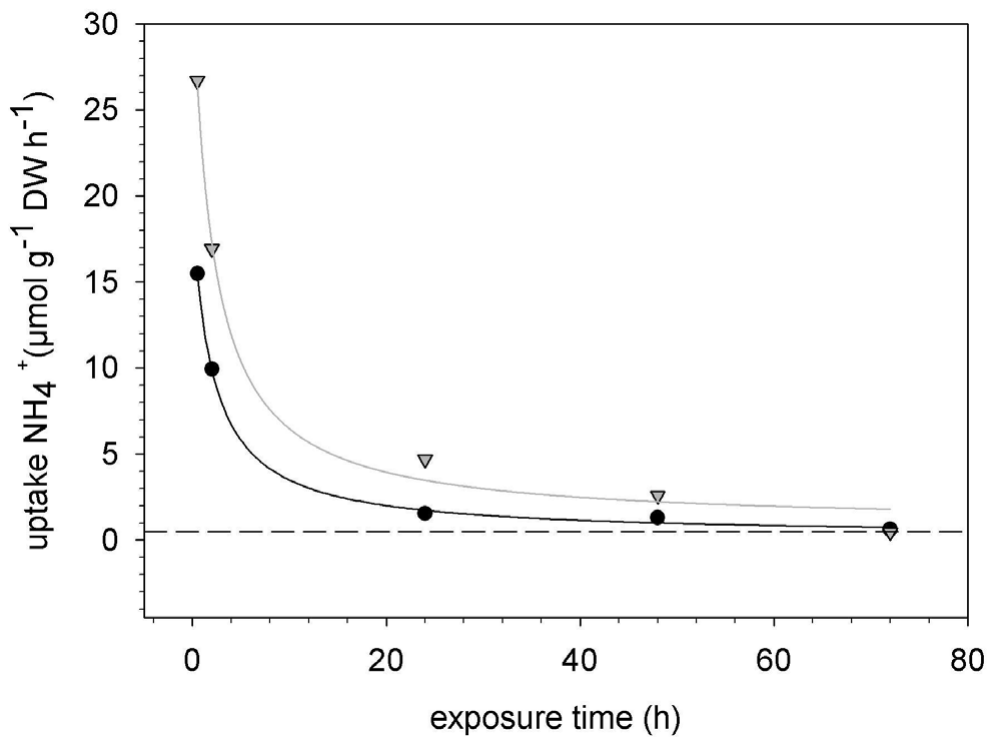
Decrease in ammonium uptake at the pristine site with increasing exposure time on. Eq. 2 fitted well (r^2^ = 0.99) for the uptake data for both 10 µM (black circles) and 100 µM (grey triangles). The half-time value ‘b’ was 2.1 hours for both concentrations. The constant (C) was lower in the 10 µM treatment (0.22 µmol N gDW^−1^ h^−1^) compared to the 100 µM treatment (0.88 µmol N gDW^−1^ h^−1^). Dashed line indicates critical N-uptake to maintain biomass production (0.8 µmol N gDW^−1^ h^−1^). Note that uptake rates at 24 hours and later were based on the depletion of ammonium in the experimental solution, whereas other rates presented in this paper are average rates calculated from ^15^N uptake.

**Figure 4:**
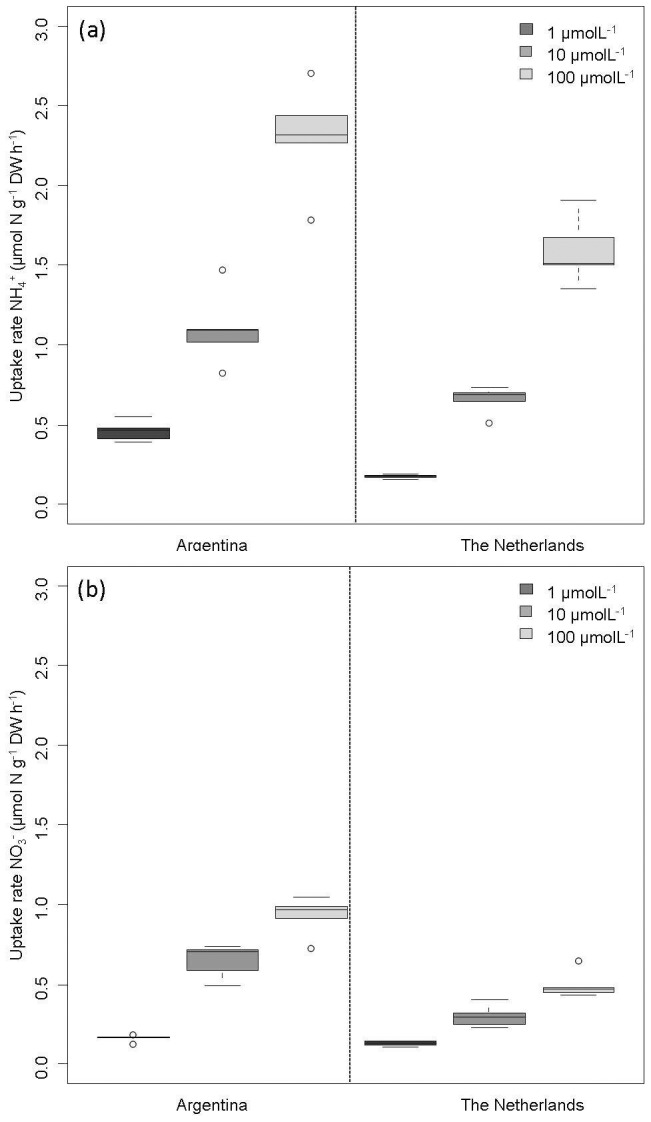
Differences in uptake rates (µmol N gDW^−1^ h^−1^) were dependent on the origin of the mosses. N-Uptake rates are shown at 1 µM (dark grey), 10 µM (grey) and 100 µM (light grey), respectively, of the pristine site (Argentina, left) and the N-polluted site (The Netherlands, right). Upper panel (a) shows ammonium (NH_4_^+^) uptake. The lower panel (b) shows shows nitrate (NO_3_^−^) uptake. Experiments lasted 72 hours.

Experiments were conducted with *Sphagnum magellanicum* (Bridel) collected from lawns at two locations with contrasting histories of nitrogen deposition. Most uptake experiments were carried out using mosses from a pristine *Sphagnum* bog (54° 45′S; 68° 20′W) in southernmost Patagonia (data in Figures 1, 2 and 3), Argentina (site description in [40], [41], where atmospheric nitrogen deposition is estimated to be as low as 1–2 kg ha^−1^ y^−1^ [10]. This site is hereafter termed the ‘pristine site’ (Figure S1 and Figure S2). The second location was a small bog in the State Forest of Dwingeloo, the Netherlands (52° 49′N; 6° 25′E; ‘N-polluted site’) with an estimated atmospheric N deposition ranging between 20 and 30 kg N ha^−1^ y^−1^, but exceeding 40 kg N ha^−1^ y^−1^ in the period 1970–2000 [42]. Mosses from this site were used in the 72 h nitrogen uptake experiments and compared with mosses from the pristine site (data in Figure 4). In April 2010, two months prior to our experiments, 10 cm thick sods of *Sphagnum magellanicum* were collected from carpets located at five distinct parts in each peatland (‘pristine site’ and ‘N-polluted site’). Summer water levels ranged between 20 cm and 40 cm below the surface at all sampling locations. Weather conditions were similar at both sites the weeks prior to sampling with high humidity and rainfall, and similar temperatures and light levels during daytime. After removing vascular plants we allowed mosses to acclimatize to lab conditions for 40 days. During this period, the water table was kept 2–5 cm below capitula (photosynthetic active apex, 1 cm long) for optimal water supply and light intensity was set at 200 µmol PAR m^−2^ s^−1^.

### Uptake experiments

Uptake rates were measured according to [39]. 25 *Sphagnum* plants (the capitulum and 20–25 mm living moss tissue below, 5 plants per container and 5 replicates per treatment) were submerged in the experimental solution containing ammonium or nitrate at different concentrations. In one treatment (0.5 h experiment) capitula and stem tissue were separated after the experiment and uptake rates were calculated separately and compared (see statistical analysis).

During the entire experiment mosses where exposed to an artificial rain solution (150 µmol l^−1^ NaCl, 30 µmol l^−1^ MgCl_2_, 30 µmol l^−1^ KCl, 10 µmol l^−1^ CaCl_2_, 10 µmol l^−1^ KH_2_PO_4_, pH 5.5–6.0) to which the different amounts of ammonium or nitrate were added. In the 0.5 h experiment we applied 1, 5, 10, 50, 100 and 500 µmol l^−1^. In the 2 h experiment we applied 10 and 100 µmol l^−1^, and in the 72 h experiment 1, 10 and 100 µmol l^−1^. Plants were pre-incubated in 20 l of artificial rain solution (N-free) for 0.5 hours before transferring them to open glass vials filled with the treatment solution. The vials were beaker-shaped and their volume was dependent on the treatment. We used volumes of 20, 5, 2, 0.5, and 0.25 litres in the 1, 5, 10, 50, 100, and 500 µmol l^−1^ treatment, respectively. To avoid concentration gradients, experimental solutions were gently stirred by using a rotary shaker (35 rpm), which clearly provided water movement around the floating mosses. In the 72 hours experiment mosses were floating in open containers of 0.3 l, in which the treatment solution was continuously replenished by peristaltic pumps (Masterflex, 7015–20; Cole-Parmer, Vernon Hills, IL, U.S.A.). Flow rates varied according to the treatment: 1 µmol N l^−1^ at 1 l h^−1^, 10 µmol N l^−1^ at 0.1 l h^−1^ and 100 µmol N l^−1^ at 0.01 l h^−1^, respectively. Theses rates rendered a nitrogen replenishment rate of 10 µmol N g^−1^DW h^−1^. The replenishment solutions were refreshed every 24 h. All treatments were replicated 5 times, and carried out at room temperature (18–21 °C) and 150 µmol PAR m^−2^ s^−1^. Mosses in the 72 hours experiment were exposed to 16 hours of light and 8 hours dark period per day.

After harvesting at the end of the experiments, mosses were carefully blotted dry, washed for 5 min in a solution of 1 M KCl added to the artificial rain (described above) and blotted dry before drying at 70°C for 48 hours. Oven dried mosses were ground in liquid nitrogen before being analysed for ^15^N and total N using isotope ratio mass spectrometry (Finnigan MAT Delta Plus, Waltham, MA, USA; see Kleinebecker *et al.*, 2009 for details). Results were corrected for background ^15^N content that was determined for each site separately (n = 10 per site). To obtain net nitrogen uptake rates we divided the uptake of ^15^N by the enrichment of N-sources used: ^15^NH_4_Cl (10 atom% ^15^N) and Na^15^NO_3_ (5 atom% ^15^N). In addition, we estimated uptake rates in the 72 hours experiment by means of depletion of nitrogen in the experimental solution related to the dry weight (cf. [43], [44]. Samples of the experimental solutions (24, 48 and 72 hours after experiment started) were directly frozen (−20°C) until colorimetrical analysis of ammonium (Traacs 800+ auto-analyzer). This allowed us to compare average uptake rates over 72 hours, determined using IRMS, with the uptake at intermediate time points (24, 48 and 72 hours after experiment started).

To estimate passive uptake, assumed to mainly represent adhesion to the cell walls, we measured nitrogen uptake in autoclaved (1 hour at 120°C) *Sphagnum* plants. After autoclaving, mosses had maintained their morphology. Following the procedure described above, plants were exposed for 0.5 hours to ^15^N-ammonium or ^15^N-nitrate at three concentrations: 1 µM, 10 µM and 100 µM, respectively. Samples were exposed to two different washing solutions: demineralised water (releasing the loosely attached N-fraction) and a 0.2 M SrCl_2_ solution (complete exchange of cations by Sr^2+^ ions).

### Uptake kinetics – effects of concentrations and increasing exposure time

We used a Michaelis–Menten kinetics combined with a linear component (k), conform earlier work on uptake by rice and corn roots [45], [46] to describe the observed uptake rates with increasing strength of the nitrogen solutions (1, 5, 10, 50, 100 and 500 µM) for mosses (n = 25) exposed for 0.5 hours. The 3 parameters were fitted to the uptake data by performing a regression analysis (single rectangular hyperbola) in Sigma Plot 10.0 (Systat Software, Inc.), using the following formula:
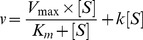
(1)where v is the measured uptake rate at a given substrate concentration [S], V_max_ is the maximum uptake rate at substrate saturation and K_m_ is the Michaelis–Menten constant (substrate concentration at which uptake occurs at half the maximal uptake, i.e. the half saturation constant). The constant k modulates the linear component in Eq.1. In Figure S3 & Table S1 of Supporting Information S1 we discuss the consequences of omitting this linear component to describe the saturation kinetics of nitrogen uptake in *Sphagnum* mosses. For clarity and comparison, both approaches are shown in figure 1, as Michaelis–Menten kinetics is more commonly applied without adding a linear component [47], [48].

We also estimated the dependency of uptake rates on exposure time t for treatments receiving 10 µM or 100 µM ammonium. Parameters for the effects of exposure time (Eq. 2) were fitted to the uptake rates at 0.5, 2, 24, 48 and 72 h by performing a regression analysis (hyperbolic decay) in Sigma Plot 10.0, using:
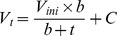
(2)where V_t_ is the uptake rate at the exposure time t, and b is the time after which V_t_ decreased to half of the initial uptake rate (V_ini_). Uptake rates at 24, 48 and 72 hours were based on the depletion of nitrogen in the experimental solution. The constant C represents the rate of background nitrogen uptake e.g. to supply nitrogen consumed by growth and natural nitrogen losses. Under field conditions the constant C may be related to “critical N-uptake”, that is the N-uptake rate needed to sustain the maximal growth rate. Aldous (2002b) estimated the annual nitrogen requirement of a growing *Sphagnum* carpet to range between 0.18 and 0.35 mol N m^−2^ y^−1^. This is equivalent to an average hourly nitrogen consumption of 0.14–0.57 µmol N g^−1^DW h^−1^ when assuming capitulum biomasses of 180–360 gDW m^−2^ and a 5-month growing season, which has been found in other studies [10], [49].

### Statistical analyses

Initial data analysis implied the fitting of multiple linear regressions, followed by a check for violation of statistical assumptions. In cases where heteroscedasticity was observed, we used linear regression with the generalized least squares (GLS) extension, which allowed us to benefit from retaining the original variance structure in the data [50], [51]. Model simplification to a minimal adequate model was based on AIC e.g. Akaike Information Criteria [52], after backward selection using the likelihood ratio test (data in Fig. 2, 4). The importance of each explanatory factor in the minimum adequate model was assessed by comparison of this model with a reduced model (with all the terms involving the factor of interest removed), using the likelihood ratio test. All analyses were performed using the ‘nlme’ package (v. 3.1, [50]) in the ‘R’ (version 2.9.2) statistical and programming environment (R Development Core Team [53]).

## Results

### Kinetics of active and passive nitrogen uptake

In plants of *Sphagnum magellanicum* from the pristine site (Patagonian bog), nitrogen uptake rates during the first 0.5 h increased with increasing nitrogen concentration. Uptake of nitrogen from the fully stirred solutions could be successfully described by saturation kinetics (Fig. 1) for both ammonium and nitrate. Mosses showed ten times higher uptake rates for ammonium (V_max_ of 28 µmol N g^−1^DW h^−1^) than for nitrate (V_max_ 2.5 µmol N g^−1^DW h^−1^). Ammonium and nitrate were taken up efficiently already at low concentrations (K_m_-values of 6.5 µM and 3.5 µM, respectively). This results in uptake rates similar to 90% of V_max_ at a concentration of 100 µmol N l^−1^. The linear component k was higher for ammonium (0.022 l.g^−1^DW h^−1^) than for nitrate (0.004 l.g^−1^DW h^−1^). We found that extending the M-M kinetics by a linear component improved the fit of the uptake curves substantially (Supporting Information S1) while the conceptual model of nitrogen uptake depended only to a small extent on the linear component (Figure S1 & Table S1 in Supporting Information S1).

Uptake rates in living mosses appeared to be independent of the washing solution used (demineralised water and SrCl_2_, respectively). Thus, the fraction bound by adsorption to the cell wall is negligible compared to the fraction taken up by the cells. Furthermore, we measured passive uptake in mosses that were autoclaved. The passive uptake was up to 2 orders of magnitude lower than the uptake in living mosses. In general, autoclaved mosses absorbed only 1 to 5% (*n* = 50) of the nitrogen taken up by living mosses, with the exception of the 100 µM nitrate treatment where this fraction is 11 to 16% (*n* = 10). Uptake in autoclaved mosses increased with nitrogen concentration of the experimental solution and was highest in the ammonium treatments. Comparing the two washing solutions showed that 60–75% of ammonium was removable with SrCl_2_ in autoclaved plant material. The fraction washed-off by SrCl_2_ may indicate all ammonium bound to adsorption sites. This effect of the washing solution was not found for living mosses.

### Effect of increasing exposure time

Nitrogen uptake rates clearly decreased with exposure time. This decrease was most pronounced for ammonium. The concentration dependency found after 0.5 hours (Fig. 1) was also observed when the duration of the experiment was increased to 2 hours and 72 hours, respectively (Fig. 2) Uptake rates were, however, much lower with increasing exposure time (p<0.001, Table 1). Uptake rates after 2 hours were 1.6 times lower for ammonium, but only 1.2 times lower for nitrate at both concentrations (10 µM and 100 µM). During 72 hours uptake rates of ammonium drastically decreased, and were 6–14 times lower than rates after 0.5 hours. In contrast, nitrate uptake efficiency was less affected by exposure time and only lowered by factor 3. Consequently, the preference of ammonium uptake over nitrate uptake declined with increasing exposure time. Tissue nitrogen remained below 990 µmol N g^−1^DW in treatments with lower uptake rates than the 100 µM NH4-treatment. Interestingly, uptake rates were independent of tissue nitrogen at the start of the experiment (indicator for N saturation), which ranged from 632–921 µmol N g^−1^DW.

**Table 1:** Minimal adequate models^1^ used for data presented in Fig. 2.

Uptake rate	Factor	Log L Ratio	p-value^2^
NH_4_^+^	Concentration	58.34	**<.0001**
	Exposure time	109.40	**<.0001**
	Concentration×Exposure time	22.69	**<.0001**
NO_3_^−^	Concentration	42.06	**<.0001**
	Exposure time	50.86	**<.0001**
	Concentration×Exposure time	13.43	**<.0001**

^1^ Nitrogen saturation of moss tissue was not a significant factor.

^2^ p-values represent the importance of the explanatory factor assessed by a comparison of the minimum adequate model with a reduced model using the likelihood ratio test.

We investigated the uptake dynamics of ammonium in more detail during the 72 hours experiment by measuring ammonium depletion in the experimental solution. During the first 2 hours, uptake rates slowed down below 60% of the initial uptake rates (Fig. 3; half time value corresponds to 2.1 hours). The decline of ammonium uptake rates with increasing exposure time can be well described (r^2^ = 0.99) by equation 2 (Fig. 3). Background nitrogen consumption (C in Eq. 2) ranged from 0.22–0.88 µmol N g^−1^DW h^−1^, which was similar to nitrate uptake rates over 72 h.

### Long-term adaption to increased nitrogen supply

Mosses from the pristine site (Patagonia; Figure S1 and Figure S2) appeared to be more efficient in rapidly taking up ammonium and nitrate than mosses from the N-polluted site (the Netherlands). 40–160% higher uptake rates were observed in all treatments in the 72 hours experiment with the exception of the 1 µM nitrate treatment (Fig. 4). Both sites showed a concentration effect as described above (Fig. 2). For the 1 µM nitrate treatment, mosses from the pristine site had similarly low rates as mosses from the polluted site. This resulted in an interaction effect between site and concentration (Table 2, df = 1, *F* = 4.35, *P* = 0.047). Mosses from the pristine site were more efficient in taking up ammonium at concentrations below 100 µM, than mosses from the N-polluted site. This difference was most pronounced at very low ammonium concentrations (1 µM). At 1 µM ammonium mosses from the pristine site showed average uptake rates of 0.46 µmol N g^−1^DW h^−1^ which is still within the range of ‘critical N-uptake rates’ (Figs. 3, 4). Mosses from the N-polluted site were less efficient at concentrations of 1 µM ammonium or nitrate, resulting in total uptake of <13 µmol N g^−1^DW over 72 h. Such a low uptake is on average lower than ‘critical N-uptake rates’. In mosses from the N-polluted site, tissue nitrogen concentrations remained below 800 µmol N g^−1^DW during the experiment, showing no signs of N-saturation.

**Table 2:** Accession effect: linear models of data presented in Fig. 4.

Uptake rate	Factor	F	p-value
NH_4_^+^	Concentration	180.33	**<.0001**
	Origin	24.39	**<.0001**
	Concentration×origin	3.03	**0.0936**
NO_3_^−^	Concentration	45.36	**<.0001**
	Origin	20.91	**<.0001**
	Concentration×origin	4.35	**0.0469**

### Comparison of N-uptake in apical tissue and stem tissue

**Figure 5:**
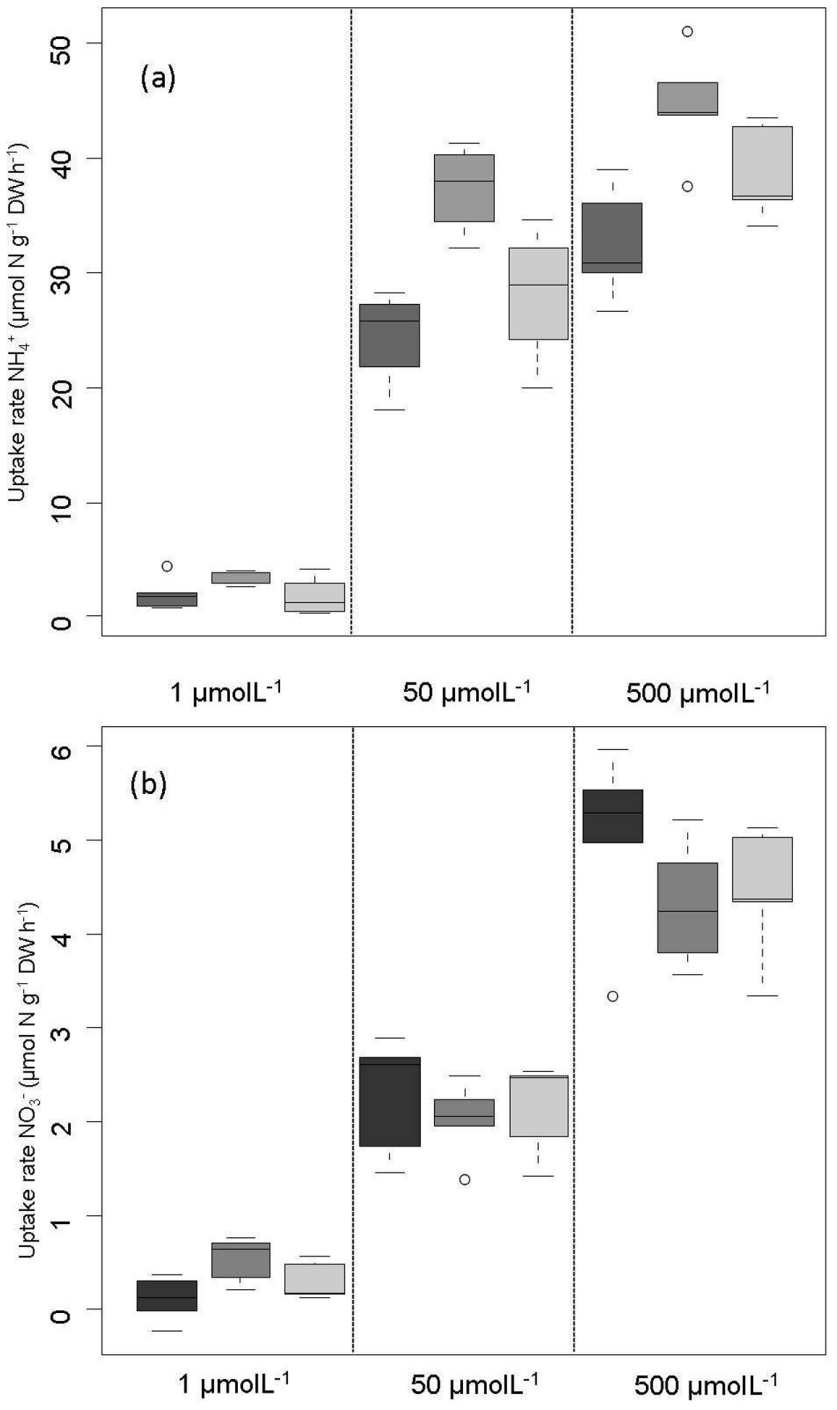
Differences between average uptake rates (µmol N gDW^−1^ h^−1^) after 0.5 hours in different fractions of *Sphagnum magellanicum* from the pristine site. We analysed capitula and stems separately, while whole plant values represent the DW weighted mean of both fractions.

As rainwater usually infiltrates deeper than the upper 5–10 mm where the capitula are situated, a larger part (30–35 mm) of the *Sphagnum* mosses were used in the uptake experiments. Lower stem tissue is likely to be in contact with soil moisture containing ammonium and nitrate for longer periods of time. We found 30% higher uptake by stems than by capitula for ammonium treatments with high concentrations (50 µM and 500 µM) (Fig. 5, Table 3). In the 1 µM nitrate treatment the specific uptake rate of stems was not significantly higher than that of capitula, differing only 0.5 µmol N g^−1^DW h^−1^ (Fig. 5, Table 3).

**Table 3:** Differences between uptake rates in capitulum and stem tissue, as shown Fig. 5.

Uptake rate stem vs. capitula	concentration (µmol l^−1^)	Factor (uptake capitula vs. stem)	T	df	p-value
NH_4_^+^	1	ns	2.762	4	0.0507
	50	0.66	4.952	4	**0.0158**
	500	0.73	6.125	4	**0.0036**
NO_3_^−^	1	0.07	5.955	4	**0.0040**
	50	ns	−1.418	4	0.2291
	500	ns	−1.939	4	0.1245

The statistical values are the result of T-tests. Significant differences are printed in bold.

### Conceptualised model of dose–exposure time relationship

**Figure 6:**
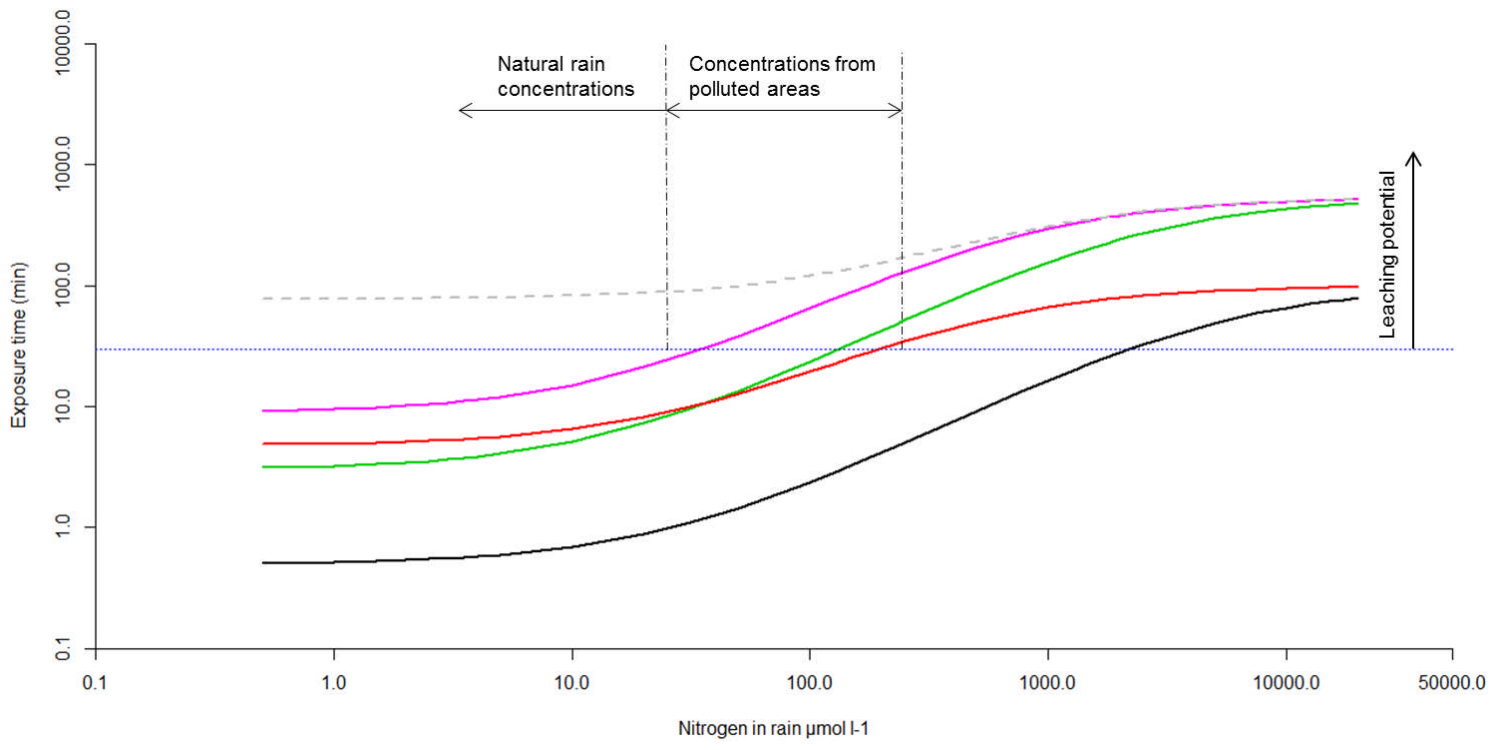
Exposure time of mosses to nitrogen increases non-linearly at increasing nitrogen concentrations. We used a conceptualized relationship (log-log) between nitrogen concentration in rain and exposure time for a living *Sphagnum* layer that would need to retain some 90% of the nitrogen load. The area below the horizontal dots (average residence time of rain) indicates an increasing potential of nitrogen leaching through the living layer of mosses. Maximum uptake rates V_max_ differ between scenarios: black “ammonium pulse” 28 µmol N gDW^−1^ h^−1^, green “ammonium long” 2.8 µmol N gDW^−1^ h^−1^, red “nitrate pulse” 2.5 µmol N gDW^−1^ h^−1^, purple “nitrate long” 0.8 µmol N gDW^−1^ h^−1^ and grey dashed line “nitrate low affinity” 0.8 µmol N gDW^−1^ h^−1^, respectively. The model was parameterised with a K_m_ of 6.5 µM for ammonium and a K_m_ of 3.5 µM for nitrate. The “nitrate low affinity” was calculated with a K_m_ of 35 µM. An increasing potential of leaching is expected above the dotted black line that indicates the upper limit of residence time of rain. Average nitrogen concentrations in rain (µmol N l^−1^) relate to yearly wet deposition of nitrogen (kg ha^−1^) by a factor 0.1 (yearly rainfall 750 mm).

In figure 6 the nitrogen uptake kinetics found were used to evaluate the efficiency of *Sphagnum* plants to retain the nitrogen deposited by rain episodes under field conditions. To estimate the exposure time to nitrogen needed by *Sphagnum* mosses to retain 90% for different nitrogen deposition loads, the following assumptions were made: a rainfall of 5 l m^−2^ (5 mm) and a living moss biomass of 500 gDW m^−2^, which is equivalent to an average bulk density of 10 gDW l^−1^ in the upper 5 cm (cf. [54], [55]). As a conservative estimate we assumed that the entire moss biomass contributes to nitrogen uptake. The nitrogen pulse was diluted 4 times by the water content in *Sphagnum* mosses that often exceeds 10 times the dry weight of mosses [56], [57]. The resulting concentrations were used in Eq. 1 to calculate uptake rates per g DW biomass. Five different scenarios were used for maximum uptake rates (V_max_) based on the present uptake experiments (Fig. 1, 2): 1) ammonium pulse exposure 28 µmol N g^−1^DW h^−1^, 2) ammonium long exposure 2.8 µmol N g^−1^DW h^−1^, 3) nitrate pulse exposure 2.5 µmol N g^−1^DW h^−1^, 4) nitrate_long exposure 0.8 µmol N g^−1^DW h^−1^ and nitrate_low affinity 0.8 µmol N g^−1^DW h^−1^. The half saturation constant (K_m_) was 6.5 µM for ammonium and 3.5 µM for nitrate. For the scenario “nitrate low affinity” we calculated using a K_m_ of 35 µM. Nitrogen concentrations in rain (µmol NH_4_NO_3_ l^−1^) are related to yearly loads of nitrogen (kg ha^−1^) by a factor of 0.105, assuming 750 mm rainfall.

Initial uptake rates of an “ammonium pulse” (0.5 h uptake rates, Fig. 1) resulted in the lowest time to remove 90% of nitrogen from rain. Since the exposure time linearly decreases with V_max_, the highest exposure times were found for the scenarios with lowest V_max_ (“nitrate long” and “nitrate low affinity”). For the scenario “nitrate long” simulations suggest that even at natural nitrate concentrations the exposure time may exceed 30 minutes, which is close to the upper limit of the average residence time of rain (dotted line in Figure 6). The simulations suggest that all scenarios except “ammonium pulse” are prone to nitrogen leaching to deeper soil/peat layers when concentrations of nitrogen are at levels typical for polluted areas.

Rain N concentrations below 10 µmol N l^−1^ (natural unpolluted rain) had surprisingly little influence on exposure time (Fig. 6). In contrast, exposure time increased substantially at N concentrations in rain above 30 µmol N l^−1^ (rain from polluted areas). A high half-saturation constant K_m_ (viz. low affinity to nitrogen) increased exposure time especially at low concentrations (uppermost curve in Fig. 6 and “nitrate pulse” vs. “ammonium long”). Nitrogen doses used in many field application experiments (>2000 µmol N l^−1^) require exposure times up two orders of magnitude higher than the residence time of rain, which may probably result in deep infiltration of nitrogen.

An increased frequency of intense rain storm events can reduce residence time of rain below ten minutes during these events. Based on the conceptualised model proposed here, we expect an increased leaching potential of nitrate but also of ammonium in case *Sphagnum* will be exposed for several hours to ammonium. Highest leaching is expected shortly after dry spells, for two reasons. Firstly, our data shows passive uptake rates to be 10 to 20 times lower than active uptake. Secondly, the water holding capacity of *Sphagnum* stands is drastically reduce due to hysteresis occurring during drying-rewetting cycles.

## Discussion

To our knowledge, this is the first study that investigates the interactions between exposure time and nutrient concentration for the uptake of ammonium and nitrate by *Sphagnum magellanicum*, which is an important peat-forming species in peatlands globally. High uptake rates found in the present study suggest almost complete, immediate (within minutes) uptake of ammonium and nitrate from rain (Fig. 6). Long-term uptake rates (Figs. 2, 3) seemed to be sufficient to maintain N-provision for growth (critical N-uptake rates). Higher uptake rates were found for stem tissue as compared to capitula tissue (Fig. 5). Stems showed a higher surface area per dry weight, which increased the relative surface of stems to the nitrogen solution. Nitrogen uptake by *Sphagnum magellanicum* from the pristine Patagonia site seemed to be much more efficient at low concentrations (K_m_ = 11 µM) than arctic *Sphagnum* spp. ([47]; K_m_ = 1001 µM) or tropical non-*Sphagnum* mosses ([48]; mean K_m_ = 59 µM for 10 species). The extremely high uptake efficiency found in the present study may reflect the nutrient-deprived conditions in Patagonian bogs and serve as a benchmark for studies in polluted areas.

The highly efficient nitrogen uptake, however, provides a serious risk of over-saturation when mosses become exposed to high atmospheric nitrogen loads and consequently high nitrogen availability in rainwater and surface moisture. Porewater concentrations of dissolved inorganic nitrogen are often in the range of 5–50 µmol N l^−1^ at low to average nitrogen deposition sites [49], [58], [59], with ammonium being the dominating species due to the low pH in *Sphagnum*-dominated ecosystems (low nitrification rates). Our studies revealed substantial uptake rates for this range of porewater concentrations. It has been shown for *Sphagnum* mosses that photosynthetic rates decrease at nitrogen tissue contents above 930 µmol N g^−1^DW [37]. Mosses would exceed their optimal nitrogen content (930 µmol N g^−1^DW) within 36 hours (extrapolating 0.5 hour uptake rates at 10 µM ammonium (15.5 µmol N g^−1^DW h^−1^) and a nitrogen tissue content of 400 µmol N g^−1^DW. Evidence for supra-optimal N-uptake was found in the 100 µM ammonium treatment over 72 h (Fig. 2). Detoxification of excess nitrogen, especially ammonium, is slow and requires both energy and products of photosynthesis (production of N-rich amino acids; [1], [60], [61]. It is therefore very likely that a mechanism for rapid down-regulation of N-uptake has evolved in *Sphagnum* to prevent excessive uptake of nitrogen and energy loss. We have indeed found such evidence of a decrease in uptake efficiency upon enhanced exposure time to nitrogen (Figs. 2–4).

### Exposure time effect

Exposure time significantly decreased nitrogen uptake rates at all N doses (Figs. 2, 3). This exposure time effect also increased with nitrogen dose (Table 1). We expected uptake rates to remain constant or to decline only modestly at low nitrogen doses (i.e. low uptake rates) as earlier studies assumed a fixed upper threshold of nitrogen uptake, which is related to nitrogen saturation of moss tissue [12], [35]. In contrast to these expectations, the present study suggests a decrease in uptake efficiency independent of tissue nitrogen concentration but present along the entire range of total nitrogen uptake (12–166 µmol N g^−1^DW) over 72 h (Fig. 3). Nitrate uptake efficiency also decreased, but substantial differences were only found at the scale of hours to days (Fig. 2). The observed reduction in nitrate uptake occurred at the same time scale as the reported decrease of nitrate reductase activity, the key enzyme in nitrate assimilation [1], [62]. A lower activity of nitrate reductase may cause an accumulation of symplastic nitrate [35] possibly resulting in down-regulation of transporter activity.

Interestingly, uptake rates decreased also at low concentrations of nitrogen and consequently resulted in low total uptake. We hypothesise that the observed rapid reduction in ammonium uptake (40% in the first 2 h; Fig. 3) is related to the saturation of temporal storage pools (vacuole, cell wall, amino acid production). Once these pools are saturated, ammonium may be taken up at rates similar to nitrogen consumption in biomass production and amino acid/protein synthesis. Little is known, however, about the kinetics of amino acid metabolism in *Sphagnum* mosses. A slow accumulation of glutamine has been reported at rates of 35 µmol g^−1^DW over 72 hours [1]. Also [61] found activity of glutamine synthetase substantially lower than ammonium uptake rates in *Sphagnum* mosses. Biomass production, the most important long-term sink of nitrogen, sequesters on average 0.1–0.6 µmol N h^−1^ per gram growing apex (capitulum) when converting seasonal to hourly growth rates. These critical N-uptake rates are in stark contrast to the actual nitrogen uptake rates observed during the first two hours, but seem to be in agreement with N-uptake rates found during the 72 h experiment (Figs. 3, 4).

The cation exchange at the cell wall could represent additional ammonium storage. The results with autoclaved mosses suggested, however, that only <1 µmol NH_4_ g^−1^DW could be adsorbed to the cell wall when mosses where exposed to a medium containing 260 μeq l^−1^ competing cations (see methods). This low adsorption of ammonium is in agreement with other studies [63], [64]. The capacity to adsorb ammonium has been shown to be lowered by competing cations like K^+^, Na^+^ and Ca^2+^ [64].

### Global change increases the risk of nitrogen leaching

Adaption to high nitrogen loads may not only occur at the scale of hours, but also after years or decades of increased nitrogen deposition [38]. Our results indeed provide evidence that the N-uptake efficiency of *Sphagnum magellanicum* decreases by a factor 1.4–2.6 in mosses that experienced long-term exposure (decades) to high nitrogen deposition (Fig. 4). A decrease of nitrogen retention would be exacerbated when *Sphagnum* moss density is reduced by nitrogen [10], [54], [55]. Therefore, we expect a substantially lower nitrogen retention by stands of *Sphagnum* from polluted sites compared to pristine mosses, which may further enhance nitrogen leaching in N polluted sites, even when nitrogen deposition would decrease. Further studies are needed to unravel the physiological mechanisms causing the reduction in uptake efficiency.

We propose a conceptual model (Fig. 6) that relates uptake efficiency to both dose and exposure time. Our model suggests enhanced leaching (lower effective retention) with increasing N loads. The model explains the leaching found in gradient studies [17], [65], pulse loading studies [34], [49], [54] and fertilisation studies [14], [26], [66]. The decrease in uptake efficiency of mosses (Fig. 2) within 72 hours results in substantially higher exposure times (Fig. 6), by which both ammonium and nitrate are at increased risk of leaching even at natural nitrogen concentrations in rain. Substantial leaching of nitrate may also occur at the short residence times associated with continuous rainfall, typical for the large oceanic bogs of North and South America [67], [68].

The nitrogen uptake kinetics found here also suggest that climate change may interact with N-retention efficiency (Fig. 6). Excessive rainfall and extended dry spells may become more frequent, and in combination will result in rapid and deep infiltration of rainwater and solutes [32], [33], [69]. Dry conditions may also lower uptake efficiency as a result of low metabolic activity and growth of mosses after desiccation [70], [71]. N-loads typically applied in field addition experiments (>1000 µmol N l^−1^; 5–40 kg N ha^−1^ per application) would remain available for days to weeks in the living *Sphagnum* layer (Fig. 6), as has been found in field studies [10], [72]. At exposure times of days, availability of applied nitrogen depends largely on hydrological changes (dilution by rain, infiltration), which may partly account for the high variation found in nitrogen uptake by *Sphagnum* mosses [21].

## Conclusion

*Sphagnum* mosses have developed a rapid uptake mechanism to deal with low availability of nitrogen, where other species in ombrotrophic environments have dealt with the same constraint by becoming insectivorous or particularly nutrient-conservative [73]. Nitrogen uptake of *Sphagnum* appears to be highly efficient (“N-sprinter”) and very well adapted to natural rain events (1–20 µmol N l^−1^), traits that have been selected for during the evolution of this 34–102 million year old genus [74]. However, *Sphagnum* has to deal with a delicate trade-off between preventing potential adverse effects of high N-uptake (Fig. 2), and promoting the competitive strength of vascular plants by substantial leaching (Fig. 6) and accumulation of nitrogen in the rhizosphere [12], [75], [76]. This trade-off extends across several scales, from cell to peatland system. In case *Sphagnum* biomass decreases, leaching of nitrogen is strongly increased [17]. Once the accumulation of nitrogen promotes the expansion of vascular plants [26], [28], *Sphagnum* growth is impeded by shading and desiccation at the ecosystem scale [7], [11], [77].

The present study indicates that long-term nitrogen retention is tightly linked to nitrogen assimilation via biomass production of *Sphagnum* mosses (Fig. 3). Productivity of *Sphagnum* mosses is often limited by water availability ([78] and literature therein) and we therefore expect a strong interaction between moisture availability and nitrogen retention. The expected hydrological extremes (e.g. [79]) as a result of climate change increase the negative effects of high nitrogen availability and can thereby stress *Sphagnum*-dominated vegetation under already low nitrogen loads. Therefore, both types of global change in concert (climate change and increased anthropogenic N input) are expected to severely change the functioning of *Sphagnum*-dominated peatlands, and complicate the conservation and restoration of these self-regulating ecosystems [80].

## Supporting Information

Figure S1 **Photo that provides an overview of the pristine site in Patagonia where material was collected from 5 plots.**
(JPG)

Figure S2 **Photo showing a close-up of a dense stand of** ***Sphagnum magellanicum*** **at the pristine site before sampling.** Note that vascular plants are scattered at a low density accounting for less than 1% of the total biomass.
(JPG)

Supporting Information S1 **Comparing two kinetic models (Michaelis-Menten vs. model used in the main text) revealed that differences between a 2-parameter model and a 3-parameter model are small within the range of natural nitrogen concentrations in rain.**
(DOCX)
