# Supplementary figures and images for: Sphagnum Mosses - Masters of Efficient N-Uptake while Avoiding Intoxication
Source: PLoS One. 2014 Jan 9;9(1):e79991. doi: 10.1371/journal.pone.0079991 (PMC3886977; doi:10.1371/journal.pone.0079991)

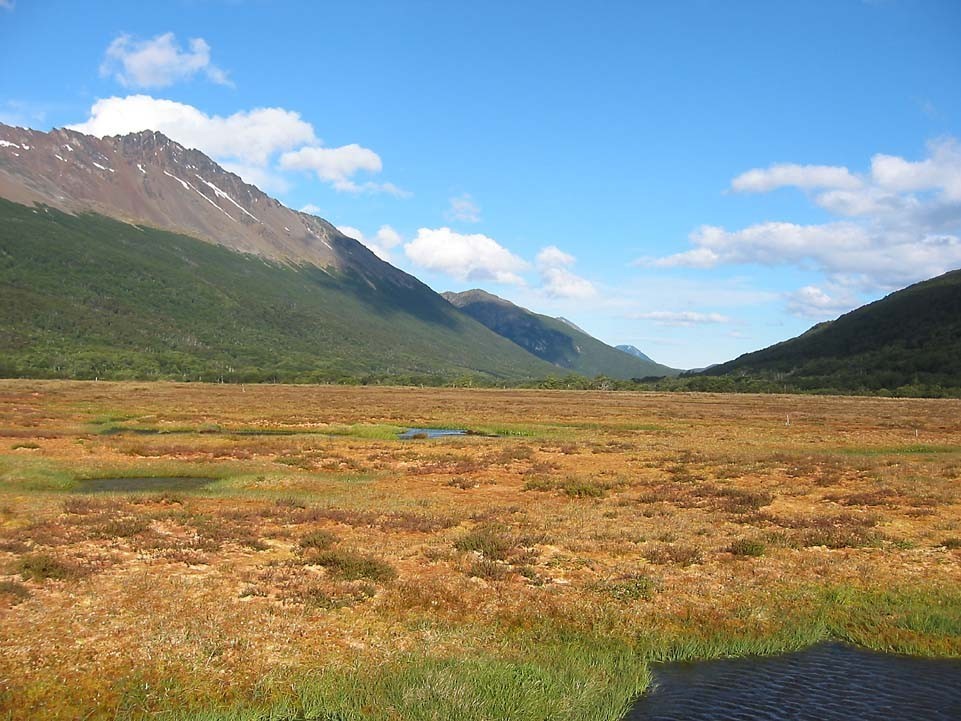

Supplement: Figure S1 — Photo that provides an overview of the pristine site in Patagonia where material was collected from 5 plots. (JPG) [file pone.0079991.s001.jpg]

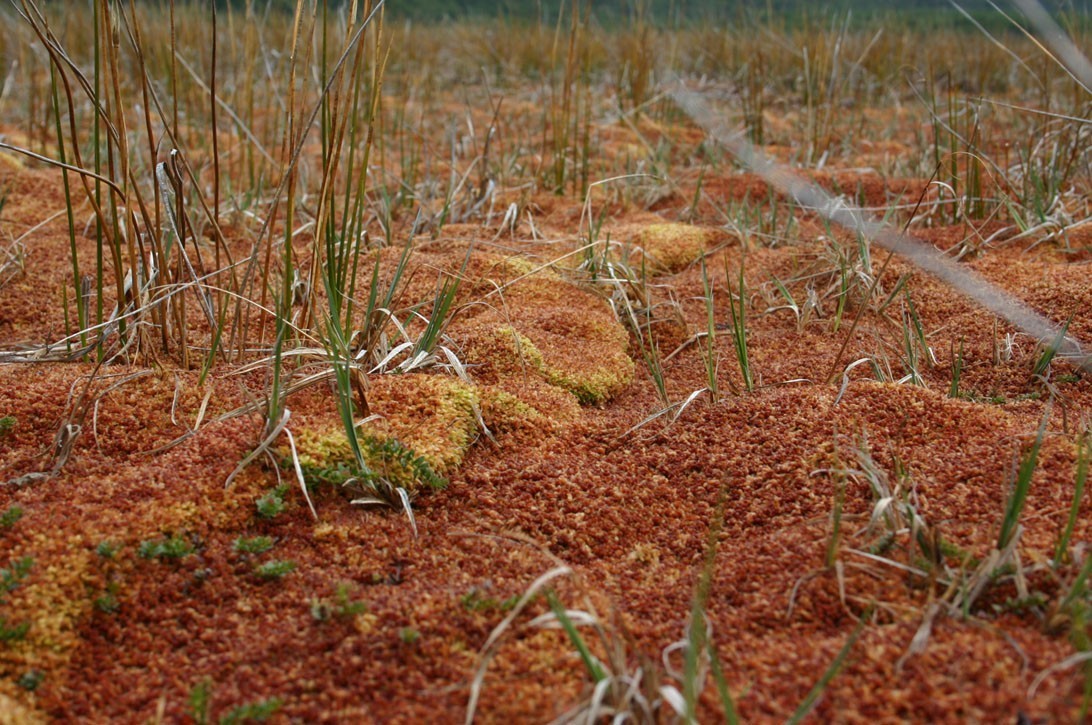

Supplement: Figure S2 — Photo showing a close-up of a dense stand of Sphagnum magellanicum at the pristine site before sampling. Note that vascular plants are scattered at a low density accounting for less than 1% of the total biomass. (JPG) [file pone.0079991.s002.jpg]
