# Supplementary material for: Sphagnum Mosses - Masters of Efficient N-Uptake while Avoiding Intoxication
Source: PLoS One. 2014 Jan 9;9(1):e79991. doi: 10.1371/journal.pone.0079991 (PMC3886977; doi:10.1371/journal.pone.0079991)
Supplement: Supporting Information S1 — Comparing two kinetic models (Michaelis-Menten vs. model used in the main text) revealed that differences between a 2-parameter model and a 3-parameter model are small within the range of natural nitrogen concentrations in rain. (DOCX) [file pone.0079991.s003.docx]

**S 1 – Parameters of uptake kinetics**

In this section we discuss the consequences of using 2 or 3 parameters to describe nitrogen uptake kinetics in (*Sphagnum*) mosses. To our knowledge most studies on nitrogen uptake kinetics in mosses have used a 2-parameter Michaelis-Menten equation (Eq. 3) [16, 39, and 47]. The first component, V_max_, describes the plateau at which uptake rates level off for high nitrogen concentrations. The second component (also termed ‘half saturation constant’), K_m_, stands for the substrate concentration at which uptake occurs at half of the maximal uptake V_max_. However, a large body of experimental data suggests that uptake rates can still increase at high concentrations (e.g. uptake rate curves that keep trending upwards with increasing substrate concentration) literature [45, 46].

|  | (Eq. 3) |
| --- | --- |

From Figure 1 (main document) it can be inferred that an uptake curve (rectangular parabolic) using 3 parameters describes the observed uptake rates (r²=0.97 for nitrate and r²=0.98 for ammonium) better than curves based on a 2-parameter Michealis-Menten approach (r²=0.81 for nitrate and r²=0.93 for ammonium) as reported in Table S1.

Table S1: Comparison of fits based on AIC values of equation 1 (3-parameters) and equation 2 (simpler, 2-parameters). The comparison was performed in Prism 6 ® Graphpad software. Comparing fittings based on a square root of residuals also preferred a fit based on Eq. 1.

|  |  |  |
| --- | --- | --- |
| Comparison of Fits | Ammonium uptake | Nitrate uptake |
| Simpler model | MM-2 Parameters (Eq. 3) | MM-2 Parameters (Eq. 3) |
| Probability it is correct | 1.81% | 0.02% |
| Alternative model | 3 Parameters (Eq.1) | 3 Parameters (Eq.1) |
| Probability it is correct | 98.19% | 99.98% |
| Ratio of probabilities | 54.17 | 5130.80 |
| Preferred model | 3 Parameters (Eq.1) | 3 Parameters (Eq.1) |
| Difference in AICc | 7.984 | 17.09 |

There are several factors that may have increased uptake rates linearly related to the medium substrate concentration. Firstly, depletion of nitrogen took place in the hyaline cells and around the leaf surface of the *Sphagnum* mosses despite mixing of the solution by stirring. In the case of insufficient mixing diffusion of nitrogen species may become a limiting factor on uptake rates. However, diffusion coefficient of ammonium (1.74 10^-5^ cm^2^.s^-1^) is almost similar to the diffusion coefficient of nitrate (1.67 10^-5^ cm^2^.s^-1^) whereas the linear component k (Eq. 1) is almost one magnitude higher for ammonium compared to nitrate.

Secondly, higher uptake rates at higher concentration may be an artefact due to the extent and the intensity of the washing process. Even though mosses where taken out of the solution immediately after 30 min it took several minutes until the mosses where washed thoroughly. During the washing period nitrogen concentration in the hyaline cells may have been still high, especially in the treatments with ammonium concentrations of 100 and 500 µmol l^-1^. Ammonium as a cation is easily adsorbed to cell walls which makes more difficult to remove during the washing process (Clymo 1963, Brehm 1968). This delay may have extended the time plants where exposed to nitrogen by several minutes.

Thirdly, linear component of ion uptake has been associated with low affinity transporters. Unfortunately, the lack of studies on *Sphagnum* mosses restricts further speculations on the importance of complex sets of ion transporters in the nitrogen uptake of Sphagnum mosses.

**Comparison of models**

The conceptualised model (Fig.6) was tested for its sensibility to changes in number of parameters used. Figure S3 (below) suggests small differences between models in a range from 10 µM to 5000 µM. Large differences have only been found for high concentrations (milimolar range) where the linear component in Eq. 1 dampens increasing exposure times whereas the classical 2-parameters Michaelis-Menten (Eq. 3) approach has already reached the plateau of V_max_.

The work presented here suggests that both estimations of uptake kinetics may be used for simulation within a range of nitrogen up to a few milimols. Latter facilitates the comparison with other studies.


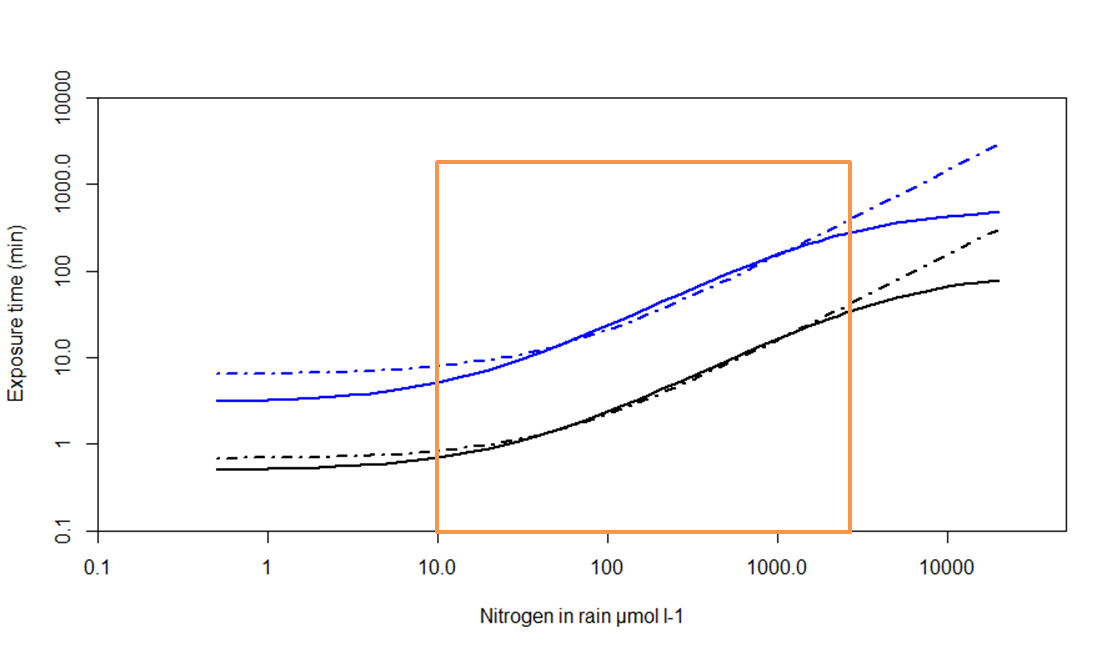


Supplementary Figure S3 compares simulated exposure times of different models calculating the uptake kinetics. For solid lines the curves were calculated using 3 parameters and the dash dotted lines were calculated using 2 parameters similar as in Figure 1. Black solid line shows the simulation for ammonium (V_max_ = 28 ; K_m_ = 6.5; k = 0.022 ) against the dash-dotted black line ammonium (V_max_ = 35; K_m_ = 11.3 ). Above these curves we plotted the blue solid line (V_max_ = 2.5; K_m_ = 3.5; k = 0.004 ) against the dash-dotted blue line nitrate (V_max_ = 3.7; K_m_ = 11.0). The orange box represents the range of nitrogen concentration found in rain water from pristine and polluted areas as well as artifical rain used in N-addition experiments.
